# Combining the Strengths of MS and NMR in Biochemometrics: A Case Study on *Buddleja officinalis*

**DOI:** 10.1021/acs.jnatprod.4c00847

**Published:** 2024-11-06

**Authors:** Andreas Wasilewicz, Alexander Areesanan, Benjamin Kirchweger, Sven Nicolay, Eva Waltenberger, Mehdi A. Beniddir, Carsten Gründemann, Judith M. Rollinger, Ulrike Grienke

**Affiliations:** † Division of Pharmacognosy, Department of Pharmaceutical Sciences, Faculty of Life Sciences, 31260 University of Vienna, Josef-Holaubek-Platz 2, 1090 Vienna, Austria; ‡ Translational Complementary Medicine, Department of Pharmaceutical Sciences, 27209 University of Basel, Campus Rosental, Mattenstrasse 22, 4058 Basel, Switzerland; § Pharmaceutical Biology, Department of Pharmacy, Ludwig-Maximilians-Universität München, Butenandtstraße 5-13, 81377 Munich, Germany; ⊥ Équipe Chimie des Substances Naturelles, BioCIS, CNRS, 439708 Université Paris-Saclay, 17 Avenue des Sciences, 91400 Orsay, France

## Abstract

Biochemometrics has emerged as promising strategy for the targeted identification of bioactive constituents from natural sources. It is based on the correlation of bioactivity data with chemical data to reveal constituents contributing to activity. Providing complementary data and structural information, MS- and NMR-based biochemometric approaches have both been separately applied in the past. The herein presented study is dedicated to the evaluation of a combined MS- and NMR-based biochemometric workflow for the unambiguous identification of bioactives. As an example, a flower extract of *Buddleja officinalis* Maxim. was selected to unravel bioactive constituents in the context of dry eye disease pathology. While NMR-based biochemometrics relies on heterocovariance analysis (HetCA) of ^1^H NMR spectra using the previously established ELINA approach, a biochemometric molecular network was generated for the MS-based approach. Both analyses were performed in parallel and were ultimately combined to increase their power to identify the bioactive constituents from the complex mixture. As a result, phenylethanoid glycosides and triterpene saponins were discovered as main contributors for the antioxidant and cytotoxic effects of the extract. This article illustrates the advantages, opportunities, and limitations of MS and NMR in the context of biochemometrics.

## Graphical abstract

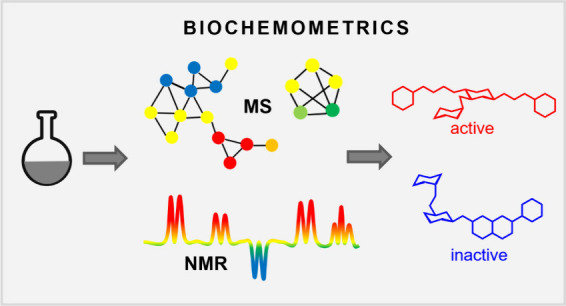

For more than a century, the isolation and identification of bioactive constituents from a multicomponent mixture has been a core discipline in natural product research. Bioactivity-guided fractionation has been the most commonly used approach that is based on the continuous separation and iterative biological testing of fractions derived from a bioactive extract. Besides being a time-consuming endeavor, bioactivity-guided fractionation rather favors the isolation of highly abundant and easily detectable constituents from a complex mixture, whereas minor bioactive constituents with poor detection might be overlooked. In recent years, a new strategy called “biochemometrics” has emerged as successful approach for the targeted and unbiased identification and isolation of bioactive constituents.^1^ In brief, biochemometrics is based on the correlation of bioactivities and chemical/metabolite profiles of multicomponent mixtures such as extracts and fractions.^2^ While bioactivity data can be derived from any assay system (cell-free, in vitro, in vivo), the chemical analysis is commonly performed by LC-MS or NMR, providing essential information for compound identification.

In order to apply biochemometrics for the identification of bioactives, a workflow called Eliciting Nature’s Activities (ELINA) was previously developed.^3^ With this approach, a bioactive extract is separated into microfractions (MFs), containing varying ratios of constituents in a consecutive series of at least three MFs, so-called packages. All MFs are tested for bioactivity, and in parallel, ^1^H NMR spectra are recorded. For selected MF packages with increasing or decreasing activity so-called heterocovariance analysis (HetCA) is performed to correlate activity data with NMR data.^4^ The resulting color-coded ^1^H NMR pseudospectrum reveals certain proton signals to be either positively or negatively correlated to activity, facilitating the identification of active and inactive constituents. Since its introduction, ELINA has been successfully applied in several studies^5–7^ and has also demonstrated its potential to reveal synergistic activities of constituents within complex mixtures.^8^

Besides the ^1^H NMR-based biochemometrics, the ELINA workflow also includes LC-MS to complement compound annotation/identification within the bioactive MFs. In fact, MS-based biochemometric and metabolomic approaches are more common in literature than NMR-based approaches. In particular, molecular networking became a powerful tool for the visualization and organization of MS/MS data of compounds from complex mixtures, allowing the identification of new compounds, the dereplication of known metabolites and the discovery of bioactive constituents.^9,10^ In order to identify bioactives, the biological data of multicomponent mixtures (e.g., extracts and fractions) can be implemented in the corresponding molecular network to prioritize the (phyto)­chemical workup for the targeted isolation of putatively active constituents.^11,12^ In more advanced bioactivity-based molecular network approaches, the results from biochemometric analyses are integrated to the molecular network, allowing to pinpoint to molecules/nodes with positive correlation to bioactivity.^13^

Both, MS- and NMR-based biochemometric approaches exhibit their respective advantages and limitations. Regarding sensitivity, MS is superior to NMR, which can be crucial for the identification of minor bioactive constituents. On the other hand, NMR provides universal and quantitative detection, while MS detection depends on a compound’s ability to ionize. In a previous study,^14^ which applied both MS- and NMR-based biochemometric methods nonsimultaneously, the potential complementarity of these two techniques was hypothesized and was challenged in the herein presented work.

In order to validate the potential of a combined MS- and NMR-based biochemometric approach with an authentic biological sample, we selected a recently investigated extract^15^ from the flowers of *Buddleja officinalis* Maxim. (BO-LLE) which was evaluated for its beneficial effects in the context of dry eye disease (DED). The flower buds of *B. officinalis* have been used in Traditional Chinese Medicine, particularly for the treatment of eye disorders such as blurred vision, photophobia and eye swelling which are symptoms associated with DED. Based on this traditional application, *B. officinalis* flowers have gained attention as potent herbal drug for DED treatment.^16^ Thereby, BO-LLE showed antioxidant, wound healing and immunomodulatory properties but also moderate cytotoxic effects in a human corneal epithelial cell-transformed (HCE-T) cell line.^15^ The ELINA workflow was applied to investigate the constituents contributing to these activities. For the NMR biochemometrics, a HetCA analysis was conducted. In order to enable a direct comparison between MS and NMR results, a biochemometric molecular network approach was established in parallel and integrated into the ELINA workflow. The biochemometric results of both analyses were simultaneously evaluated to determine the bioactive constituents of the investigated extract (Figure 1).

**Figure 1:**
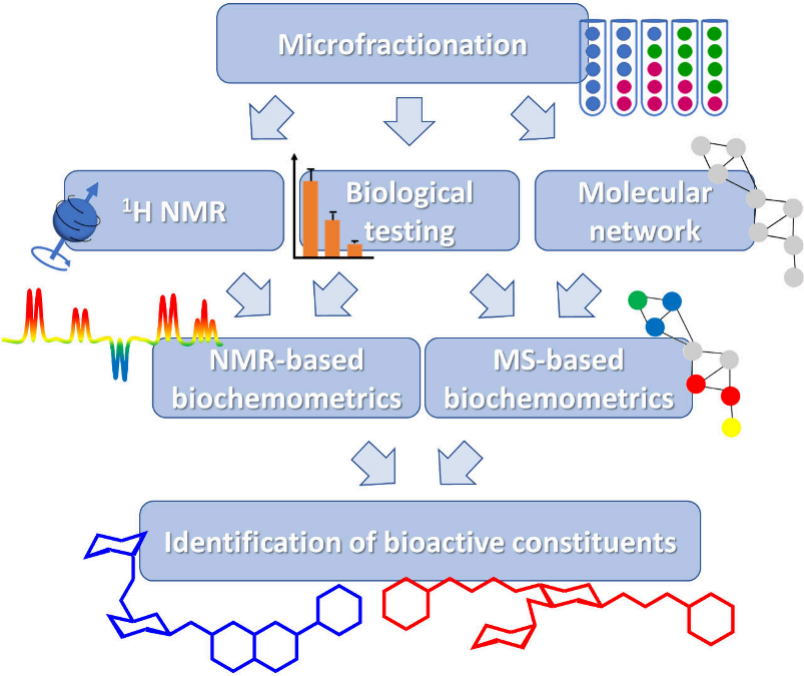
Overview of the applied biochemometric approaches, starting with the microfractionation of a bioactive crude extract and exploiting MS- and NMR-based biochemometrics. The obtained MFs are tested for biological activity; in parallel, ^1^H NMR spectra and LC-MS^2^ data for molecular network generation are recorded. The spectral data are correlated with the biological data, revealing chemical features that contribute to activity (red-colored ^1^H NMR resonances in the pseudospectrum and nodes in the molecular network) and those negatively correlated with the determined activity (blue-colored signals).

## Results and Discussion

### Microfractionation of *B. officinalis* Flower Extract (BO-LLE)

In a previous work, the lead-like enhanced extract of *B. officinalis* flowers, BO-LLE, was generated and evaluated for its potential beneficial effects in the context of DED.^15^ Hereby, BO-LLE exhibited distinct and dose-dependent antioxidant activities in a cell-free 2,2-diphenyl-1-picrylhydrazyl (DPPH) assay and in an intracellular reactive oxygen species (ROS) assay using a UVB-exposed human cornea epithelial cell-transformed (HCE-T) cell line. In addition, BO-LLE revealed to be cytotoxic against HCE-T cells in the highest concentration tested (100 μg/mL). For this study, BO-LLE was selected as an example for the application of the proposed combined NMR- and MS-based biochemometric workflow to determine the constituents responsible for the antioxidant and cytotoxic activity. In accordance with the ELINA workflow, BO-LLE was first fractionated into 34 microfractions (MF1-MF34) using normal phase flash chromatography.^3^ To obtain suitable MFs for statistical correlations, the goal was to fractionate BO-LLE in such a way that a quantitative variance of constituents across several consecutive fractions was achieved. This was monitored by TLC analysis of the collected tubes before pooling them to 34 MFs based on the TLC fingerprints. For all MFs, ^1^H NMR spectra and UHPLC-MS^2^ data were recorded and their antioxidant and cytotoxic properties were determined.

### Antioxidant Properties and Cytotoxicity of Microfractions

BO-LLE and MF1-MF34 were tested for antioxidant activity in a DPPH assay. In addition, cell cytotoxicity in HCE-T cells was investigated using a water soluble tetrazolium salt (WST-1) assay. BO-LLE showed antioxidant activity in the DPPH assay with an IC_50_ of 21.97 μg/mL (Figure 2A). MF21 and MF22 revealed the strongest activity in the DPPH assay, with 77.7% and 67.1% radical scavenging capacity at 10 μg/mL, respectively (Figure 2A). While most of the MFs were well tolerated by HCE-T cells at 10 μg/mL, MF29 and MF30 as well as BO-LLE at higher concentrations (100 and 300 μg/mL) were cytotoxic (Figure 2B).

**Figure 2:**
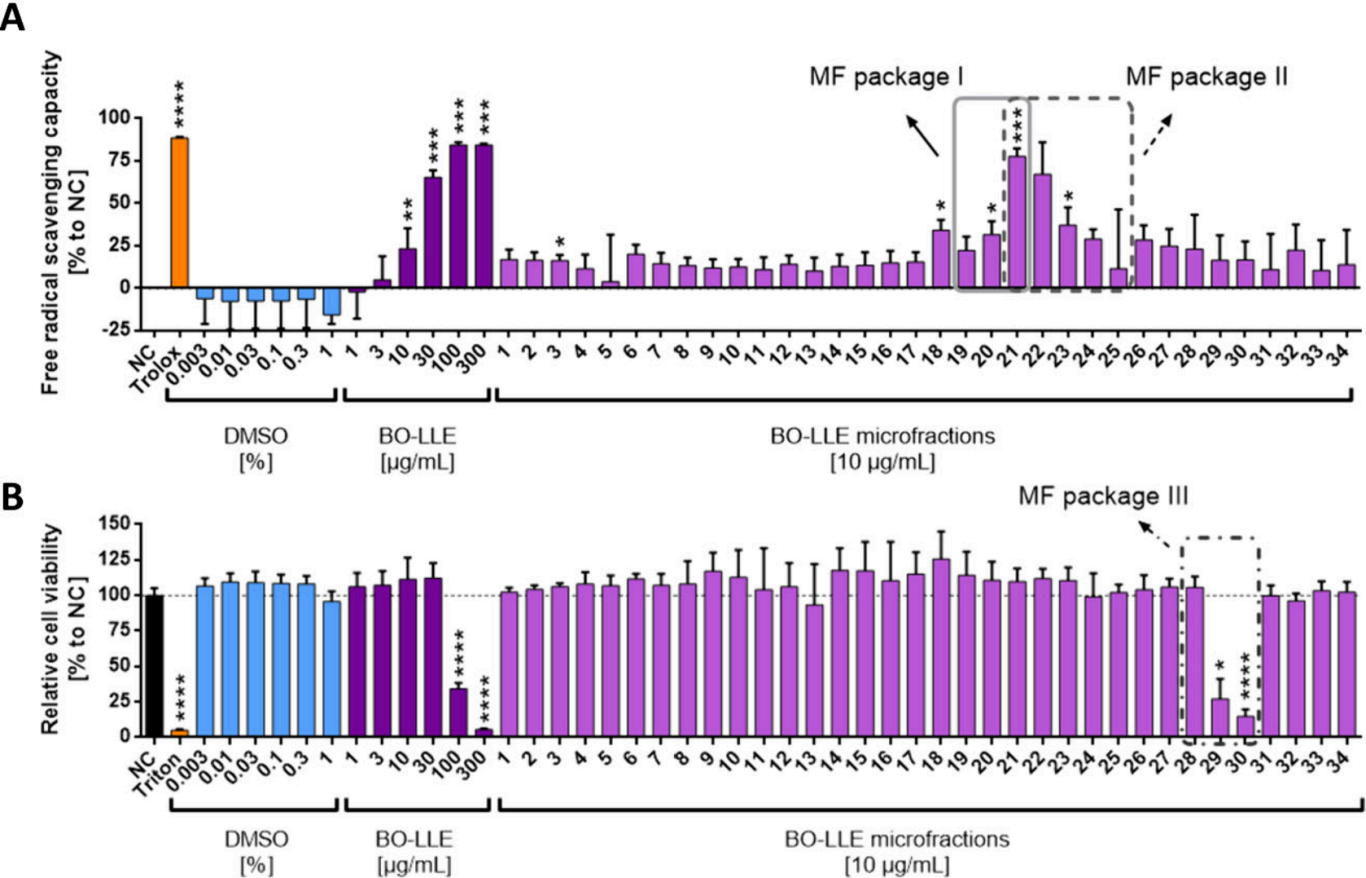
Bioactivity of BO-LLE and MFs in the (A) DPPH assay and (B) WST-1 assay. (A) In a cell-free assay, BO-LLE or MFs were incubated for 30 min with DPPH (100 μM) to spectrophotometrically quantify their free radical scavenging capability. Trolox was used as a positive control. (B) HCE-T cells were treated with BO-LLE or MFs for 24 h, and the cytotoxicity was determined by colorimetric WST-1 assay. Triton-X 100 (Triton) was used as a control. Packages I–III used for biochemometric analysis are shown as gray boxes. The data were normalized to the negative control (NC) and presented as mean ± standard deviation. *n* = 3; *p < 0.05, **p < 0.01, ***p < 0.001.

In order to unravel the antioxidant activities based on the applied DPPH assay, two packages of MFs were determined as particularly suitable for biochemometrics: package I (MF19-MF21) and package II (MF21-MF25; Figure 2A). To identify cytotoxic compounds, package III (MF28-MF30) was selected based on its stepwise increase of cytotoxicity in the WST-1 assay (Figure 2B).

### Molecular Network Generation

For the MS-based biochemometric approach, a molecular network of the obtained MFs was generated (Figure 3). After preprocessing the UHPLC-MS^2^ spectra in MZmine 3, a feature-based molecular network was generated using the Global Natural Product Social Molecular Networking (GNPS) infrastructure.^17–19^ The resulting molecular network contains 1389 nodes (= MS features) including 26 spectral families (>3 nodes). Structural annotation of nodes was conducted based on (i) GNPS experimental spectra library matches, (ii) in silico annotation by network annotation propagation (NAP)^20^ and (iii) individual MS spectra analysis, determining the putative molecular weight of a compound, combined with literature search using SciFinder (https:\\scifinder-n.cas.org) to find potential known constituents reported for *B. officinalis* flowers.

**Figure 3:**
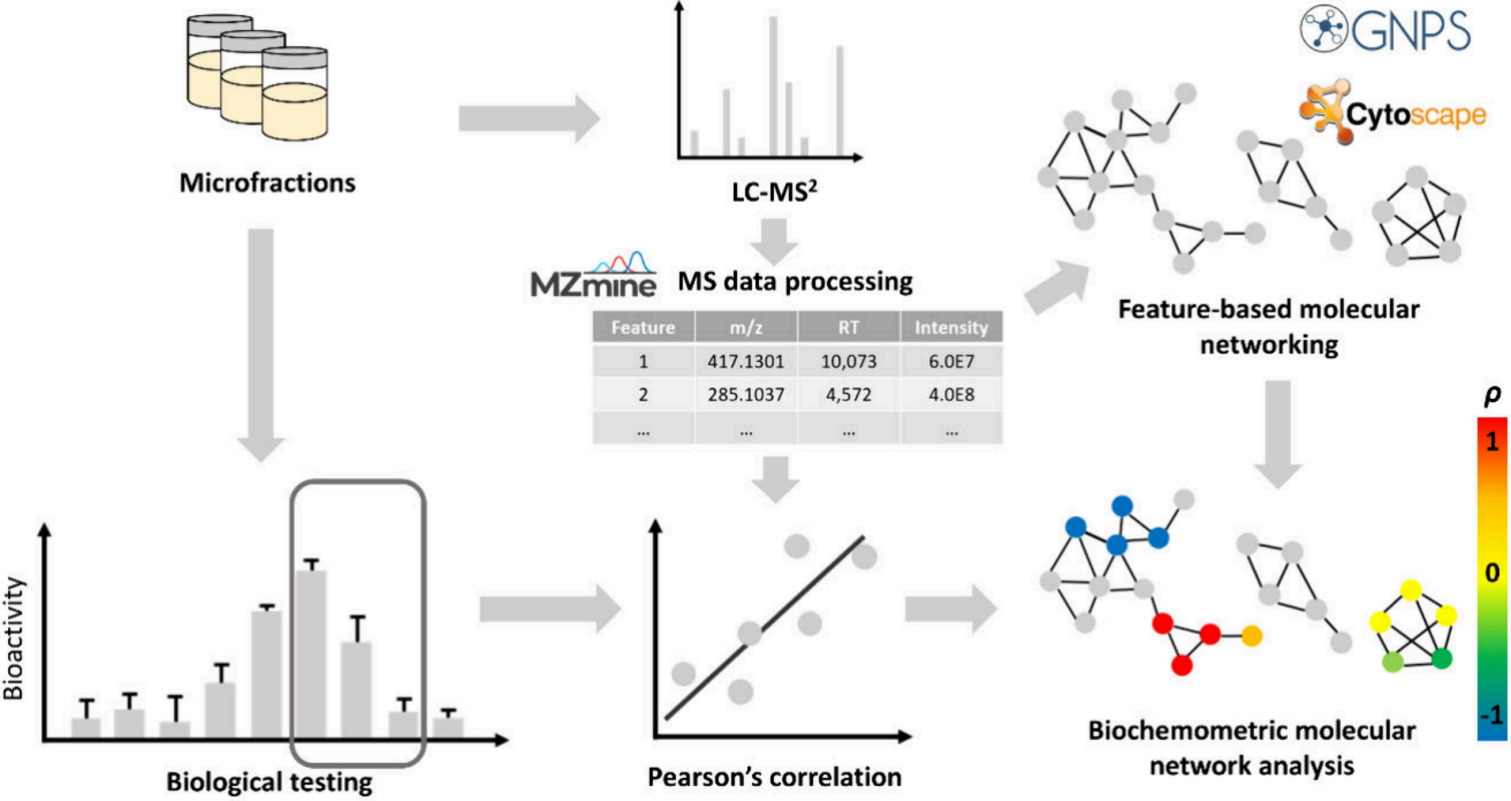
Overview of the established biochemometric molecular network approach. For all microfractions, bioactivity was determined and UHPLC-MS^2^ data were recorded, processed, and used for the generation of a molecular network. The bioactivity and MS data from a specific microfraction package are correlated by Pearson’s correlation. The resulting correlation coefficient of each MS feature (i.e., node) is integrated to the molecular network and depicted based on a color code. Nodes with positive correlation are shown in red and orange, nodes with no correlation are depicted in yellow, and nodes with negative correlation are colored in blue and green.

For adding information on antioxidant and cytotoxicity properties to the network, bioactivity data and the MS features of the selected MF packages (= detected ion with specific *m*/*z* value, intensity, retention time and MS^2^ spectrum) were statistically correlated using Pearson’s correlation which has been performed previously in a similar manner.^13^ Therefore, the MS feature intensities determined during preprocessing were correlated to the corresponding bioactivities of the MFs. The Pearson’s correlation calculation resulted in a correlation coefficient ranging from −1 to 1, whereas values close to 1 indicate a pronounced positive correlation between an MS feature and bioactivity, values around −1 refer to a negative correlation. Coefficient values of about 0 indicate no correlation. The resulting correlation coefficients obtained for each feature were integrated and visualized in the molecular network using a color code. Thereby, nodes (= MS feature) with positive correlation coefficient values are depicted in red and orange, nodes with negative values are shown in blue and green and nodes with no correlation are colored in yellow. Gray nodes refer to features of all remaining MFs which were not part of the respective fraction package and thus not included in the calculations.

### Identification of Bioactive Constituents by MS- and NMR-Based Biochemometrics

To identify the constituents contributing to the antioxidant activity, packages I (MF19-MF21) and II (MF21-MF25) were investigated. Since *B. officinalis* flowers contain phenylethanoid glycosides such as verbascoside (*syn*. acteoside) with well-known antioxidant activities in vitro^21^ and in vivo,^22^ this analysis serves as proof-of-concept for the applied combined biochemometric approach. The biochemometric molecular network analysis of packages I and II both pointed toward one and the same spectral family (family A) that showed several red- and orange-colored nodes (= positively correlated MS features), indicating to contain the constituents responsible for the activity of MF21 and MF22 in the DPPH assay (Figure 4A and 4B). The majority of these nodes were attributed to two compounds with similar retention times (RT 3.13 min and RT 3.31 min) which formed several adducts and fragments during MS ionization (Figure 5). GNPS spectra matches and in silico annotation suggested that these compounds belong to phenylethanoid glycosides. Thereby, one compound was dereplicated as verbascoside by comparison of MS data and retention time with an authentic reference. Verbascoside has also been previously reported as major constituent in *B. officinalis* flowers.^23^ The second compound was annotated as a verbascoside isomer because it possesses the same molecular formula. Nevertheless, the exact compound structure could not be established based on MS data, since there are several structural congeners such as *cis*-acteoside, isoacteoside and forsythoside A reported for *B. officinalis* flowers.^23^

**Figure 4:**
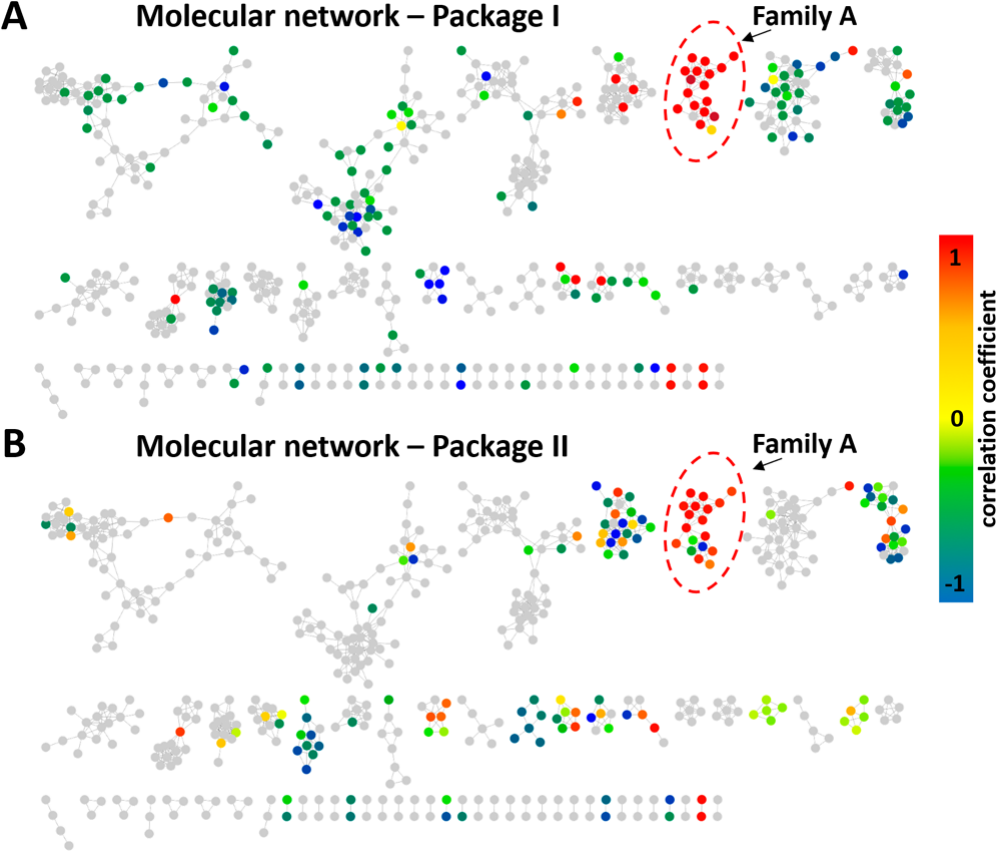
Biochemometric molecular network of (A) package I (MF19-MF21) and (B) package II (MF21-MF25) to identify constituents with activity in the DPPH assay. The color code is based on the correlation coefficient. Nodes with positive correlation coefficient values are depicted in orange and red, while negative values are shown in green and blue and nodes with no correlation are colored in yellow. Gray nodes refer to features of all remaining MFs that were not part of the respective fraction package.

**Figure 5:**
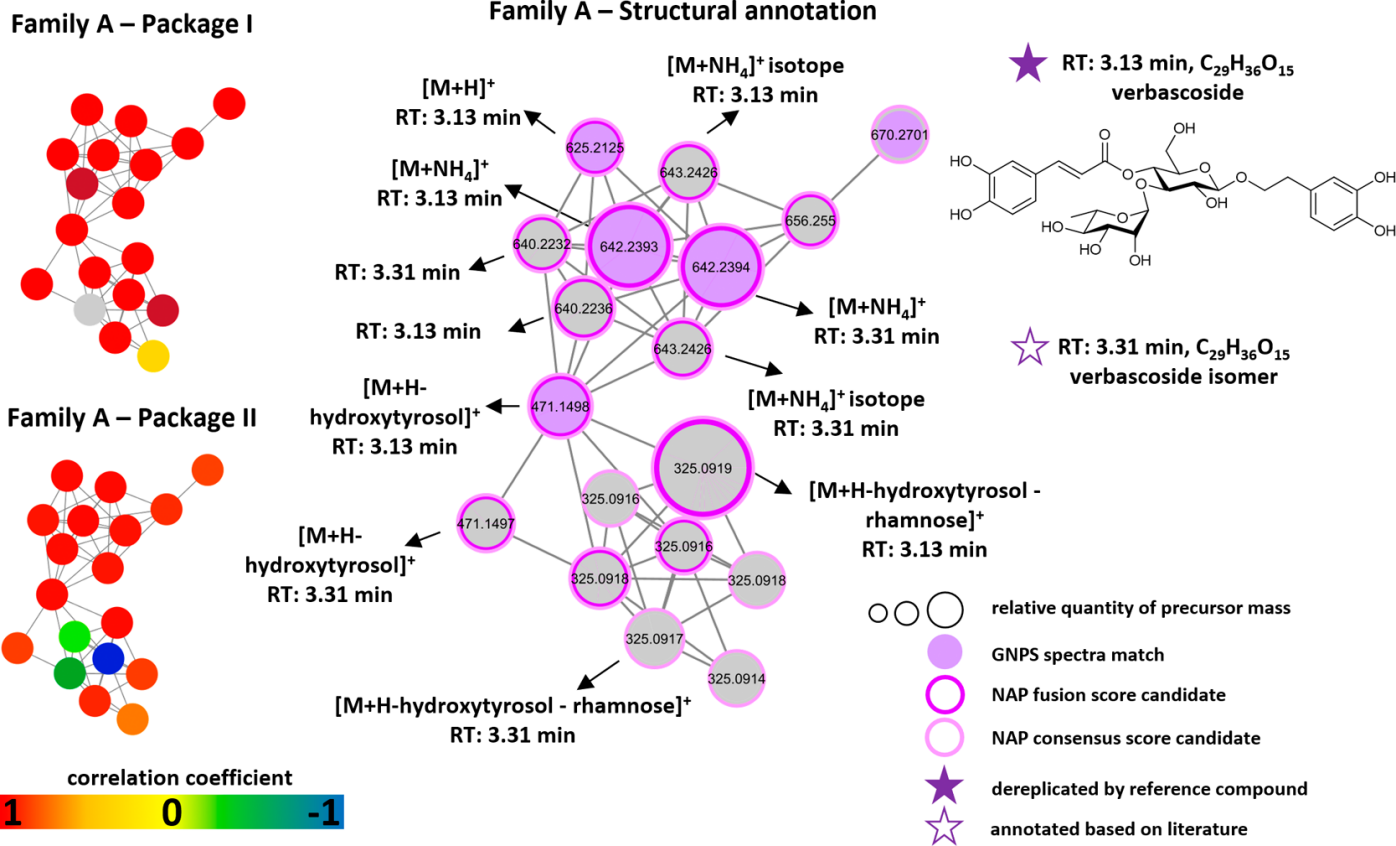
Structural annotation of positively correlated nodes of packages I and II from spectral family A. Nodes with spectral matches from GNPS experimental database are colored in lilac. Nodes with NAP in silico annotation possess borders colored in dark pink (fusion score) and light pink (consensus score). The node size indicates the relative quantity based on the MS^1^-derived precursor mass.

For the NMR-based biochemometric analysis of the ELINA workflow, HetCA analyses were performed, correlating the bioactivity and recorded ^1^H NMR spectra of the MFs from packages I and II (Figure S1). The resulting pseudospectra enabled the identification of NMR signals that are positively (red, upward signals) and negatively (blue, downward signals) correlated to activity (Figure 6A and 6B). By considering the structural information gathered from the biochemometric molecular network analysis, most of the red colored ^1^H NMR signals in both pseudospectra could be unambiguously assigned to the phenylethanoid glycoside verbascoside (Table S1) by comparison with literature data.^24^ Furthermore, four additional minor red signals in the downfield chemical shift area (δ_H_ 5.6–7.2) were visible which were attributed to aromatic/ethylenic protons, belonging to the caffeic acid residue of the second verbascoside isomer. The analysis of these proton resonances showed that two signals, at δ_H_ 5.65 and 6.82, have coupling constants of about 13 Hz, indicating a *cis*-configuration of the caffeic acid double bond (Figure 6C). Since there were no further red colored signals observed, this verbascoside isomer was identified as *cis*-acteoside according to literature.^25^ In addition, the signal intensities of the ^1^H NMR spectrum of MF21 were analyzed, revealing verbascoside as major constituent and *cis*-acteoside as a minor compound.

**Figure 6:**
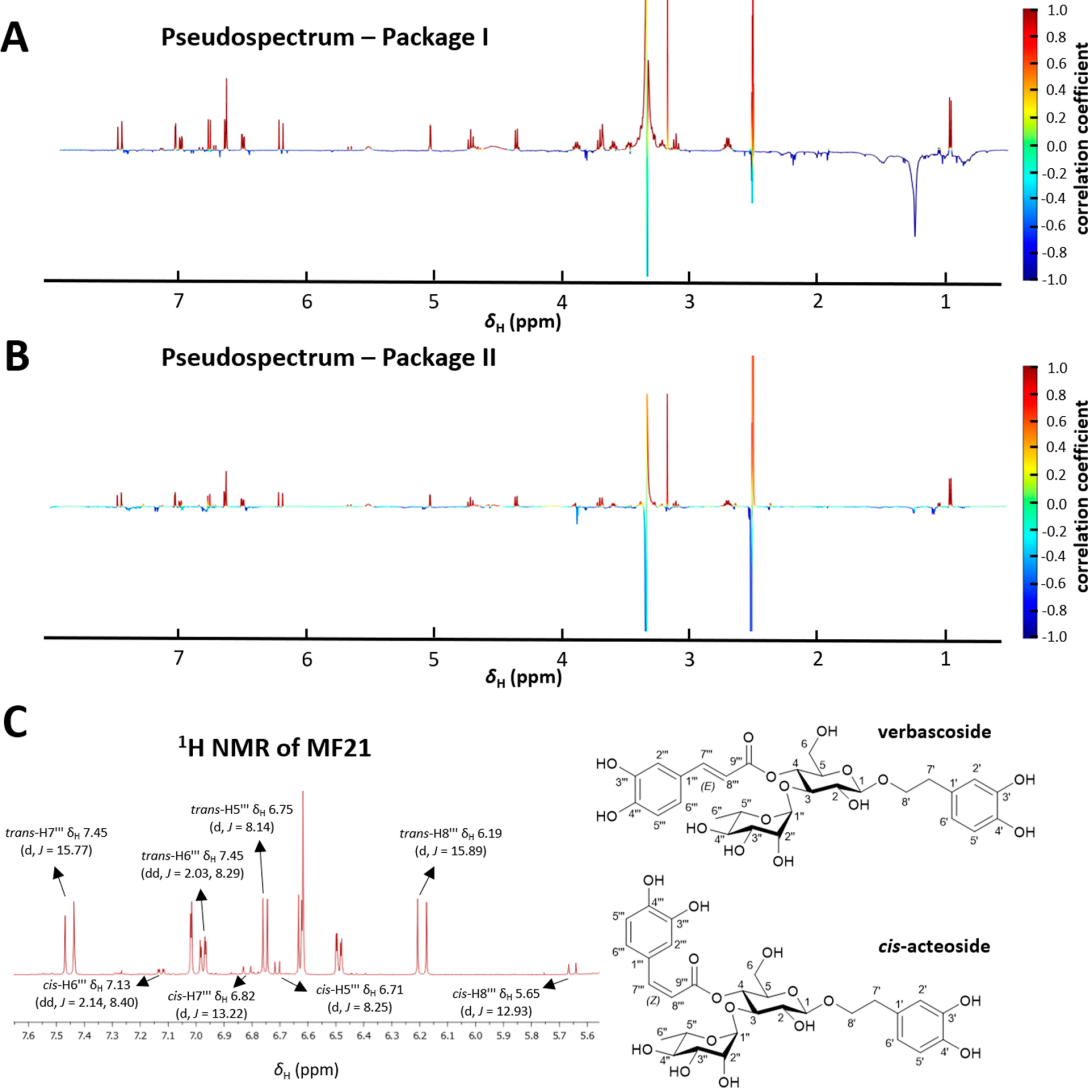
^1^H NMR pseudospectra derived from HetCA of (A) package I (MF19-MF21) and (B) package II (MF21-MF25) to identify the bioactive constituents from the DPPH assay. (C) ^1^H NMR spectrum (δ_H_ 5.6–7.6) of MF21 and chemical structures of the positively correlated constituents verbascoside (proton signals labeled as “*trans*”) and *cis*-acteoside (proton signals labeled as “*cis*”).

To validate the prediction of the combined biochemometric approach (molecular network and the HetCA analyses), the in house available verbascoside, the major compound of MF21 and MF22, was tested in the DPPH assay (Figure S2). Thereby, verbascoside showed 75% scavenging activity at 10 μg/mL (≈ 16 μM) in the DPPH assay, corresponding well to the determined activities of MF21 (77.7%) and MF22 (67.1%) at 10 μg/mL. Hence, the biochemometric approach led to the targeted identification of the bioactive constituent responsible for activity in the DPPH assay. Additionally, verbascoside showed a distinct activity in an intracellular ROS assay (29.1% of ROS level compared to untreated control at 10 μg/mL) in UVB-exposed HCE-T cells which was comparable to the results of the most active fractions MF21 and MF22 (Figure S3). Thus, verbascoside was determined as the main antioxidant agent of the investigated *B. officinalis* flower extract BO-LLE. Based on the color-coded ^1^H NMR pseudospectrum, we can further conclude that the minor constituent *cis*-acteoside additionally contributes to the antioxidant activity of BO-LLE.

For the identification of the antioxidant constituents in packages I and II, the combined MS- and NMR-based biochemometric approach indicated two bioactive constituents. Based on molecular networking, the structural class for these compounds was unveiled and their tentative annotation was facilitated. However, the exact molecular structure could not be determined, as these compounds possess the same molecular formula and identical MS^2^ spectra. Verbascoside was ultimately identified by comparison of the retention time with an authentic reference but could not be distinguished from structural congeners by MS data only. However, the complementary application of ^1^H NMR-based HetCA, facilitated the identification of the second verbascoside isomer as *cis*-acteoside as additional antioxidant principle next to verbascoside. Hence, this example shows the power of combining NMR- and MS-based biochemometric approaches for straightforward and precise structure elucidation of the bioactive constituents in mixtures. Experimental validation of the antioxidant activity of verbascoside ultimately confirmed the correct assignment of bioactivity to this constituent, highlighting the suitability of the combined biochemometric approach.

### Identification of Cytotoxic Constituents Using MS-Based and NMR-Based Biochemometrics

The molecular network analysis of package III (MF28-MF30) revealed two spectral families (family B and C) with multiple red nodes, indicating cytotoxic constituents of MF29 and MF30 (Figure 7A). Visual inspection of these families showed that some of the nodes from families B and C share the same retention time (RT 6.58 and 7.54 min) referring to the same compounds (Figure 7B). Based on in silico annotation from network annotation propagation (NAP), the nodes from family C were annotated as oleanane-type triterpene saponins. Additionally, saponins with an *m*/*z* value of 1095 [M + Na]^+^ such as the nodes identified in family C were previously reported for *B. officinalis* flowers.^26^ The corresponding nodes in family B could also be attributed to the respective aglycones of the saponins caused by fragmentation on the MS^1^ level.

**Figure 7:**
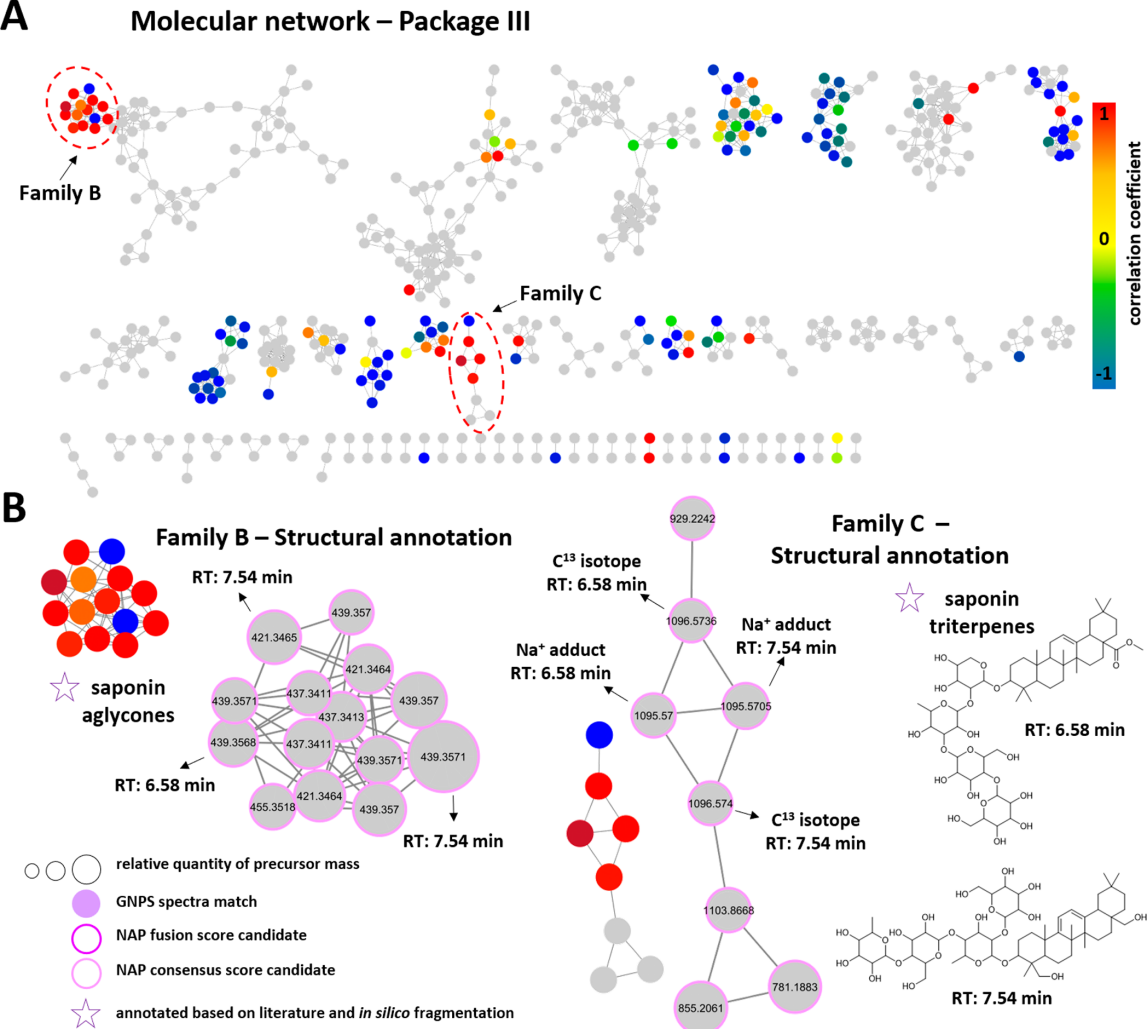
Molecular network of (A) package III (MF28-MF30) used to identify compounds responsible for cytotoxicity from the WST-1 assay in HCE-T cells. (A) The node color is based on the correlation coefficient. (B) Structural annotation of positively correlated nodes from spectral families B and C. Nodes with spectral matches from the GNPS experimental database are colored in lilac. Nodes with NAP in silico annotation possess borders colored in dark pink (fusion score) and light pink (consensus score). The node size indicates the relative quantity based on the MS^1^-derived precursor mass. The structures show the best-ranked NAP consensus score candidate for the nodes belonging to the compounds with RT 6.58 and 7.54 min.

The HetCA-derived ^1^H NMR pseudospectrum of package III showed several overlapping red colored resonances in the upfield chemical shift area which are found in the cytotoxic MF29 and MF30 (Figure 8; Figure S4). Taking the molecular network analysis into account, these aliphatic signals were tentatively attributed to the methyl groups of triterpene saponin aglycones. Moreover, a positively correlated proton signal was visible at δ_H_ 5.80 (*J* = 10.3 Hz) which could be assigned to a double bond of a triterpene aglycone.^26^ Further positively correlated signals between δ_H_ 1.2 and δ_H_ 2.0 corresponding to aliphatic proton signals of a triterpene aglycone and resonances at δ_H_ 3.0 and δ_H_ 5.0 belonging to protons of sugar residues support the assumption of triterpene saponins as potential cytotoxic constituents. In addition, analysis of the negatively correlated signals enabled to identify the flavonoid linarin and the terpenoid crocin III from which the characteristic proton signals were assigned according to literature.^27,28^ This is in accordance with the molecular network approach which indicated negative correlations for both of these constituents (Figure S5). Hence, the biochemometric analysis suggests that these two constituents are not responsible for the observed activity of MF29 and MF30.

**Figure 8:**
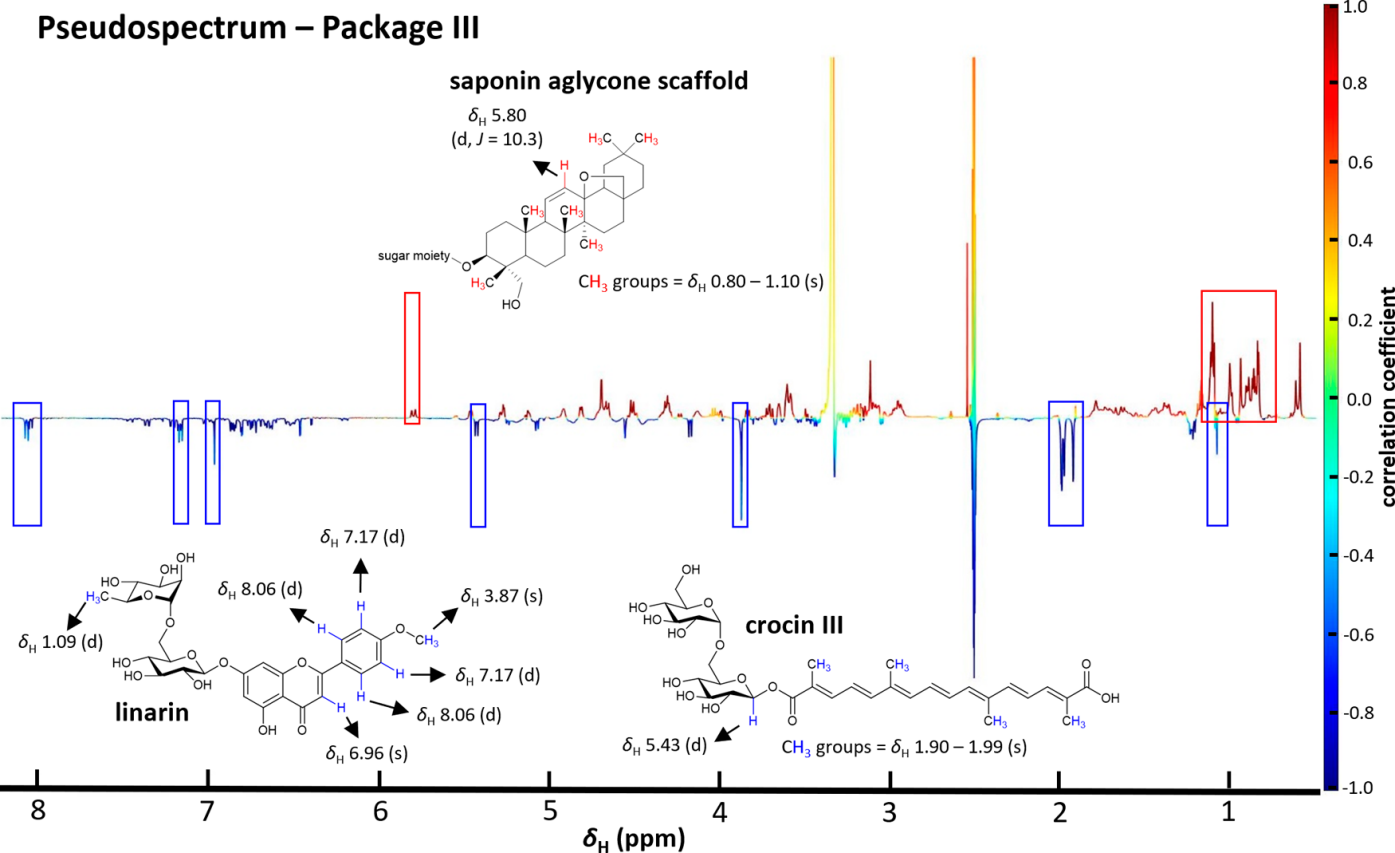
HetCA-derived ^1^H NMR pseudospectrum of package III (MF28-MF30) allows the identification of the cytotoxic as well as noncytotoxic constituents based on the WST-1 assay in HCE-T cells. Positively (red) and negatively (blue) correlated signals are assigned to the chemical structures identified in package III.

Based on this biochemometric results, targeted isolation of the potentially bioactive triterpene saponins was conducted, pursuing the identification of the constituent with the characteristic positively correlated proton signal at δ_H_ 5.80. Therefore, MF30 was first fractionated by size exclusion chromatography, resulting in a saponin-enriched fraction. For further separation, flash chromatography was conducted which led to the isolation of the targeted saponin which was unambiguously identified as mimengoside A by the interpretation of 1D and 2D NMR spectra (Table S2) and comparison to literature data.^26^ Mimengoside A has previously been isolated from the flower buds of *B. officinalis*.^26^ The comparison of the positively correlated proton resonances of package III with the proton signals of mimengoside A confirmed the successful isolation of the saponin predicted as cytotoxic by the biochemometric analysis (Figure 9). Positively correlated nodes with RT 7.54 min in the molecular network could be unambiguously dereplicated as mimengoside A.

**Figure 9:**
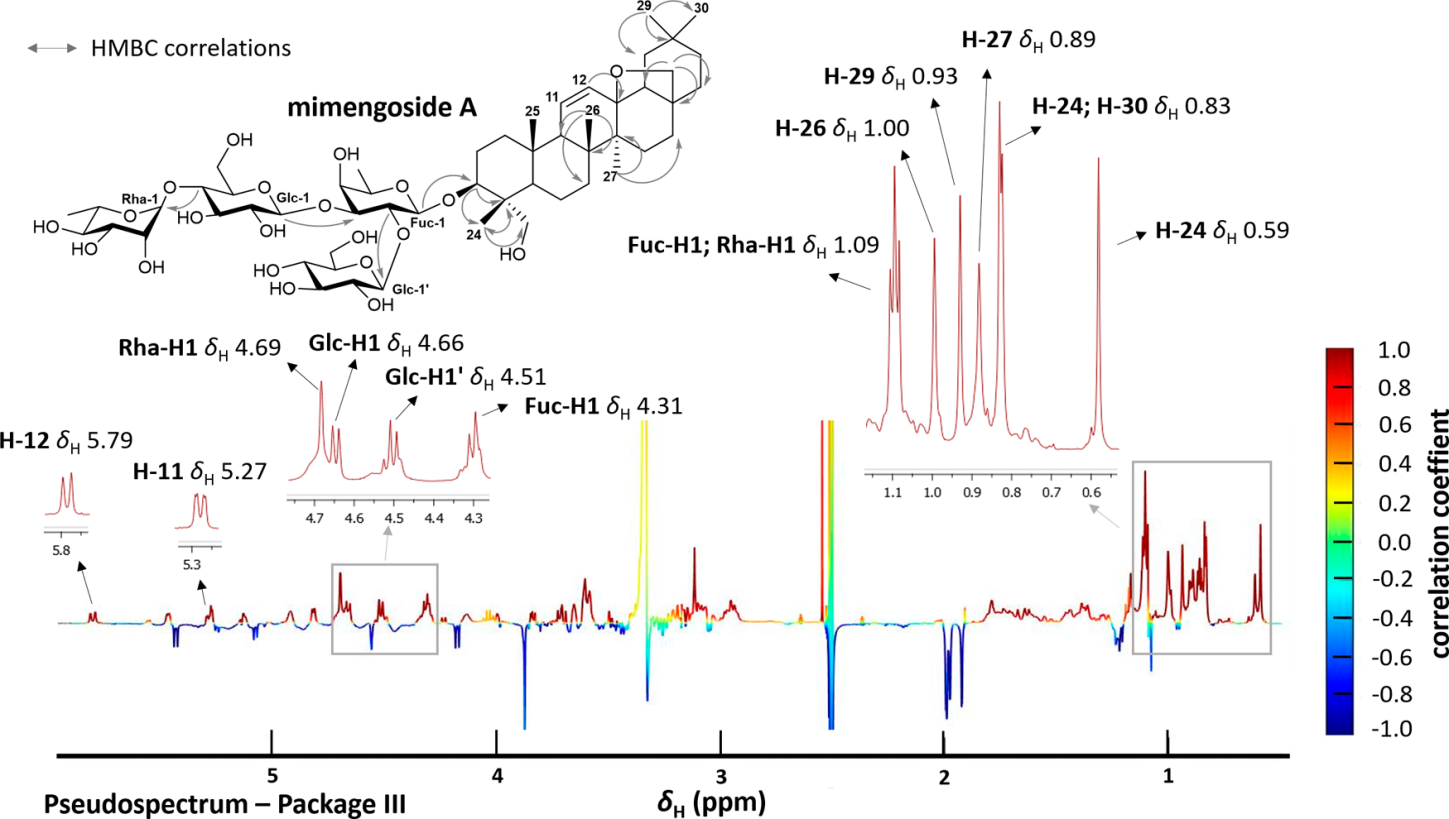
Comparison of the HetCA-derived ^1^H NMR pseudospectrum of package III with ^1^H NMR signals of the isolated mimengoside A.

In order to validate the hypothesis of the presence of the cytotoxic saponins in BO-LLE, the saponin-enriched fraction derived from MF30 was tested in the WST-1 assay (Figure S6). The viability of treated HCE-T cells decreased significantly below 25% at 10 μg/mL and higher concentrations compared to the negative control with a CC_50_ of 5.02 μg/mL. Thereby, this fraction showed similar cytotoxicity as HCE-T treated with 10 μg/mL of MF29 and MF30, confirming saponins as the cytotoxic constituents of BO-LLE at 100 μg/mL.

Both biochemometric analyses indicated triterpene saponins as compound class responsible for the cytotoxic activity in package III. Thereby, the molecular network approach again facilitated to identify the structural class of the positively correlated constituents, enabling the assignment of proton signals from the NMR pseudospectrum to the structural features of saponins.

In conclusion, over the past decade, different strategies based on MS or NMR data have emerged, harnessing the power of biochemometrics to swiftly and precisely identify and differentiate between bioactive and inactive compounds within complex mixtures.^3,13,29,30^ In this study, the established ELINA workflow, which mainly depends on ^1^H NMR-based HetCA analysis, was complemented with an MS-based molecular networking approach. Both strategies were applied to unravel active as well as inactive constituents of *B. officinalis*.

The herein presented combination of both MS- and NMR-based biochemometrics using the same set of MFs and activity data enabled to highlight the complementarity of these two techniques. Interestingly, apart from the identified active constituents, the MS^2^-based molecular network of all three investigated MF packages revealed additional red-colored nodes distributed across several spectral families, belonging to fragments of the bioactive compounds and additional constituents. These additional constituents indicated by the molecular network might also contribute to some extent to activity or coeluted just coincidentally with the bioactives during microfractionation of the extract. However, regarding the HetCA ^1^H NMR pseudospectra, corresponding red-colored proton signals were absent. This discrepancy can be explained by the higher sensitivity of MS where even very minor constituents are detected depending on instrument settings and compound ionization. In contrast, NMR with its lower sensitivity, provides universal and quantitative detection of constituents independent of their ability to ionize.

Without further information contained in the molecular network, it is challenging to assess the contribution of molecules (positively correlated nodes) to bioactivity. Nevertheless, the molecular network can provide crucial structural information, facilitating the dereplication of well-known constituents or revealing the structural class of the potential bioactives. The analysis of the positively correlated NMR features enables to determine the bioactive constituents. The quantitative information derived from NMR allows to assess the contribution of the positively correlated constituents to the observed bioactivity of the multicomponent mixture (i.e., MF package). Alternatively, semiquantitative information can be obtained by additional use of universal detectors such as ELSD or CAD.^31^

In addition, with the organization of MS features into spectral families based on similar MS^2^ spectra, molecular networking uncovers structural congeners of bioactive constituents to establish a structure–activity relationship. Independent of the annotated bioactivity correlation, this information is highly valuable to find further bioactives which might be present in too low amounts to be detected in the NMR-based approach.

In theory, biochemometric molecular networking might be superior to NMR regarding the identification of bioactive minor, low abundant ionizable constituents. However, micro fractionation as implemented in the ELINA workflow helps to overcome sensitivity limitations in NMR. In summary, the combination of both biochemometric approaches facilitates the unambiguous identification of active and inactive constituents prior to isolation.

Based on the herein performed biochemometric analyses, verbascoside and *cis*-acteoside were unraveled as the antioxidant constituents of the investigated *B. officinalis* flower extract BO-LLE. The antioxidant activity of *B. officinalis* flowers is an essential feature for the therapeutic properties against DED.^32^ Hence, phenylethanoid glycosides might be considered as reliable biomarkers for the quality assessment of *B. officinalis* containing products (e.g., eye drops) in the future. In addition, triterpene saponins from BO-LLE were identified as cytotoxic compounds for HCE-T cells. When considering a direct application of *B. officinalis* containing products on the eyes, these cytotoxic triterpene saponins should be depleted. Besides the herein investigated biological properties, additional bioactivities including anti-inflammatory and immunomodulatory effects might also play a role in the beneficial effects of *B. officinalis* flowers in the context of DED.^15^ Therefore, further studies are needed to elaborate the constituents contributing to these bioactivities, enabling the generation of optimized *B. officinalis* extracts for the treatment of DED.

## Experimental Section

### General Experimental Procedures

NMR experiments were conducted on a Bruker Ascend 500 MHz NMR spectrometer (Bruker, Billerica, MA, USA) equipped with a TCI Prodigy CryoProbe (5 mm), an Avance NEO console and a SampleJet with 96 positions for tubes without requiring spinners. Samples were measured at 298 K in DMSO-*d*_6_ (Deutero GmbH, Kastellaun, Germany) referenced to the residual nondeuterated solvent signals (δ_Η_ 2.50 ppm; δ_C_ 39.5 ppm). The resonance frequency for ^1^H NMR was 500 MHz and for ^13^C NMR 125 MHz. Standard 1D (^1^H) and gradient-enhanced (ge) 2D experiments, like COSY, HSQC, and HMBC, were used as supplied by the manufacturer.

UHPLC-MS^2^ data were recorded using an Agilent 1290 Infinity II UHPLC (Agilent, Santa Clara, CA, USA) coupled to a hybrid quadrupole time-of-flight mass spectrometer Agilent 6546 (Agilent, Santa Clara, CA, USA). MS^2^ data were obtained by using electrospray ionization (ESI) in positive mode. The ESI source parameters as well as MS detection, ionization and fragmentation parameters were set as described previously.^11^ Chromatographic analyses were performed using an Acquity BEH C_18_ column (1.7 μm, 2.1 × 100 mm) (Waters, Milford, MA, USA) kept at 40 °C. H_2_O + 0.1% HCOOH (A) and CH_3_CN + 0.1% HCOOH (B) served as mobile phases with the following gradient: 20% B at 0.0 min, 20–52% B in 6 min, 52–98% B in 5 min, 98% B for 6 min. The flow rate was set to 0.3 mL/min.

UHPLC-ELSD analysis for the purity check of mimemgoside A was conducted on a Waters Acquity H-Class system consisting of a quaternary solvent manager equipped with an automatic sample manager (8 °C) and an evaporative light scattering detector (ELSD). Mimengosid A was analyzed using H_2_O + 0.1% HCOOH (A) and CH_3_CN + 0.1% HCOOH (B) as mobile phases delivered at a flow rate of 0.3 mL/min through an Acquity BEH C_18_ column (1.7 μm, 2.1 × 100 mm) (Waters, Milford, MA, USA) kept at 40 °C. The gradient program was 5% B at 0.0 min, 5–30% B in 3 min, 30–40% B in 3 min, 40–98% B in 5 min, 98% B for 6 min. Data acquisition was conducted by Waters Empower 3 software.

Flash chromatography was performed on an Interchim puriFlash 4250 system (Montluçon, France), equipped with ELSD, PDA and a fraction collector, controlled by Interchim Software. For the fractionation of BO-LLE, an Interchim PuriFlash Silica HP column (30 μm, 120 g) served as stationary phase. *N*-hexane (A), EtOAc (B), MeOH (C) and 50% aqueous MeOH (D) were applied as mobile phases using the following gradient: 100% A for 7.5 min, 100% A/0% B to 34% A/66% B in 49.5 min, 34% A/66% B to 0% A/100% B in 10.5 min, 100% B/0% C to 90% B/10% C in 45 min, 90% B/10% C to 70% B/30% C in 25 min, 70% B/30% C to 0% B/100% C in 5 min, 100% C/0% D to 0% C/100% D in 8 min, 100% D maintained for 30 min. The flow rate was set to 46 mL/min; 20 mL were collected per tube.

For the isolation of mimengoside A, flash chromatography was conducted on an Interchim PuriFlash C_18_ HQ column (15 μm, 4 g) via dry load application. H_2_O (A) and CH_3_CN (B) were used as mobile phases with the following gradient: from 95% A/5% B to 40% A/60% B in 30 min, 40% A/60% B isocratic for 30 min, 40% A/60% B to 2% A/98% B in 30 min, 2% A/98% B isocratic for 30 min. The flow rate was set to 2.5 mL/min and 3 mL were collected per tube.

Size exclusion chromatography was performed using Sephadex LH-20 (GE Healthcare Bio-Sciences AB, Uppsala, Sweden) as stationary phase and MeOH as mobile phase (Column dimensions: 100 cm × 2 cm).

The fractions obtained from all chromatographic steps were analyzed by TLC performed using Merck silica gel 60 PF254 plates as stationary phase and two complementary solvent systems as mobile phase. TLC mobile phase system 1 consisted of CH_2_Cl_2_–MeOH–H_2_O (9:1:0.1, v/v/v) and was analyzed at visible light after derivatization with vanillin (1% in MeOH)/sulfuric acid (5% in MeOH). TLC mobile phase system 2 comprised EtOAc–H_2_O–CH_3_COOH–HCOOH (100:26:22:11, v/v/v/v). Detection of system 2 was conducted after derivatization with natural product reagent A (1% in MeOH)/polyethylene glycol 400 at UV_366_.

Verbascoside was purchased from HWI Analytik GmbH (Rülzheim, Germay) (batch no. HWI01068; purity: 86.31% determined by quantitative NMR).

### Plant Material

The flowers of *B. officinalis* (batch no. 320697) were obtained from Plantasia GmbH (Oberndorf/Salzburg, Austria). A voucher specimen (JR-20230301-A1) is deposited at the Division of Pharmacognosy, Department of Pharmaceutical Sciences, University of Vienna, Austria.

### Extraction

For the generation of BO-LLE, 1 kg of dried *B. officinalis* flowers were defatted with 12 L *n*-hexane for 48 h on an orbital shaker (GFL 3005, Burgwedel, Germany). The defatted flowers were consecutively extracted with 12 L CH_2_Cl_2_ and 12 L MeOH on the orbital shaker. The resulting extracts were evaporated (40 °C) to dryness. The MeOH extract was further processed by liquid–liquid extraction using a solvent mixture of EtOAc–*n*–butanol–H_2_O (3:2:5, v/v/v) resulting in an organic and aqueous phase, which were separated and evaporated (40 °C) to dryness. Finally, the organic phase from the liquid–liquid extraction and the CH_2_Cl_2_ extract were combined yielding 61.36 g of dried BO-LLE.

### Microfractionation of BO-LLE

Microfractions MF1-MF34 were generated using flash chromatography. In total, 8 g of BO-LLE were fractionated using dry load application in two chromatographic runs. Based on TLC fingerprints, fractions were pooled into 34 microfractions. TLC overviews are provided in Figure S7, while fraction yields are presented in Table S3.

### Biochemometric Molecular Network Generation and Analysis

UHPLC-MS^2^ data of MF1-MF34 were recorded. The obtained MS data in.d file format were converted to.mzXML files by MSConvert (ProteoWizard) before being processed in MZmine 3 (version 3.9.0).^17,33^ Methods used and parameters in MZmine 3 are described in Table S4 and a corresponding batch file is provided in the Supporting Information.^34^ The resulting .mgf and .csv files were uploaded to the GNPS Web server to perform feature-based molecular networking.^18^ In silico annotation was conducted on Network Annotation Propagation (NAP).^20^ All settings for FBMN, spectra library search and NAP are summarized in Table S5. The final molecular network was visualized in Cytoscape (version 3.10.1). The molecular network is accessible via the following link: https://gnps.ucsd.edu/ProteoSAFe/status.jsp?task=acfc7d30dc8e4f589e0751a690a780cc. MS^2^ data used for molecular network generation are accessible by the MassIVE repository (ftp://massive.ucsd.edu/v08/MSV000096015/).

Biochemometric analysis is based on Pearson’s correlation. Thereby, the quantitative data of the features from the .csv file obtained from MZmine were correlated to the bioactivity data of the MFs. For the DPPH assay results, the bioactivity data and quantification data of the packages MF19-MF21 and MF21-MF25 were used, while the data set of MF28-MF30 was used for the analysis of cytotoxicity results. The correlation coefficient values were then imported to the molecular network in Cytoscape.^35^

### ^1^H NMR Processing and Heterocovariance Analysis (HetCA)

The ELINA workflow was performed as previously described.^3^ Briefly, ^1^H NMR spectra were recorded for each MF. Therefore, aliquots of the MFs were dissolved in DMSO-*d*_6_ to obtain a concentration of 3 mg/mL. After manual baseline correction of the ^1^H NMR spectra, the previously described HetCA analysis was performed.^3^ HetCA was conducted using ^1^H NMR spectral features of MF19-MF21 or MF21-MF25 and their corresponding DPPH bioactivity data; the same procedure was applied to fractions of package MF28-MF30 and their corresponding cytotoxicity data.

### Targeted Isolation of Mimengoside A

Approximately 85 mg of MF30 were fractionated by size exclusion chromatography in two runs. One tube per minute was collected and analyzed by TLC system 2 with vanillin/sulfuric acid as spraying reagent. Based on the TLC fingerprints, the collected tubes were pooled to three fractions, including the saponin-enriched fraction (=fraction 2; 45 mg). The presence of the targeted saponins was confirmed by UHPLC-MS analysis. Next, approximately 40 mg of the saponin-enriched fraction 2 were separated by flash chromatography in reversed phase mode resulting in 88 collected tubes in total. The tubes were pooled into three fractions based on TLC analysis using solvent system 2 and vanillin/sulfuric acid as spraying reagent. Fraction 3 yielded the targeted saponin (12.86 mg) with a purity of >98% according to UHPLC-ELSD analysis as previously described.^15^ Structure elucidation was performed based on the interpretation of 1D and 2D (HSQC, HMBC, COSY) NMR data recorded using DMSO-*d*_6_ as solvent.

### Free Radical Scavenging Activity

A cell-free assay based on 2,2 diphenyl-1-picrylhydrazyl (DPPH; Sigma-Aldrich) was performed to investigate the free radical scavenging capacity of samples. Thereby, DPPH decolors to pale yellow from dark violet when reacting with hydrogen-donating antioxidant compounds. The assay was conducted based on Brand-Williams et al.’s protocol with slight modifications.^36^ In a 96-well plate, a final concentration of 100 μM DPPH was mixed into a series of the BO-LLE concentrations (1–300 μg/mL), MFs (10 μg/mL) or verbascoside (10 μg/mL) in MeOH. The vitamin E derivative, Trolox (100 μM, Tocris Bioscience), was used as a positive control. The plates were incubated for 30 min in the dark at room temperature. Afterward, the absorbance was measured spectrophotometrically at 517 nm (TECAN infinite M Plex) and the scavenging capacity was calculated as using the following formula, where blank is the absorbance of the reagent without the test extract:

Free radical scavenging capacity(%)=(blank−sample)/blank

### Cell Culture of HCE-T Cells

The SV40-immortalised human corneal epithelial cell-transformed cell (HCE-T) line was acquired from the Riken Cell Bank (RCB2280) Araki-Sasaki et al.^37^ and cells were cultured as described by Ziniauskaite et al.^38^ with DMEM/F-12 medium (Sigma-Aldrich), supplemented with 5% FCS (BioConcept), 5 μg/mL insulin, 10 ng/mL human epidermal growth factor (EGF), 0.5% DMSO, and 1% penicillin-streptomycin (all from Sigma-Aldrich) and incubated at 37 °C with 5% CO_2_. Cells were subcultured when at 85% confluency using 0.05% trypsin-EDTA solution (Thermo Scientific). Prior to conducting an experiment, cells were inoculated in a 96-well plate (2 × 10^4^ cells/well), incubated for 24 h, and changed to a phenol red-free nonsupplemented nonserum medium for 24 h.

### Cell Viability Assay for HCE-T Cells

HCE-T cells were cultivated as described previously.^15^ The viability of the treated HCE-T was determined based on the relative mitochondrial activity of viable cells to convert the tetrazolium salt (WST-1; Sigma-Aldrich) into formazan. HCE-T cells were incubated with, BO-LLE (1–300 μg/mL), saponin-enriched fraction (both 0.3–300 μg/mL) or MFs (10 μg/mL) for 24 h at 37 °C with 5% CO_2_. The cells were washed with PBS before were incubated with 1:10 of WST-1 for 2 h at 37 °C, then were measured spectrophotometrically at 450 nm with 650 nm as a reference reading (TECAN infinite M Plex). Triton X-100 (1% v/v, Sigma-Aldrich) was used as a positive control.

### Intracellular ROS Measurement

Based on the principle of cell-permeable fluorescein, 2′,7′-dichlorodihydrofluorescein diacetate (H_2_DCFDA; Invitrogen), that emits fluorescence upon oxidation by oxidants, intracellular ROS measurement was performed as described by Abenǵozar-Vela et al.^39^ and Katsinas et al.^40^ with slight alterations. HCE-T cells were incubated with 25 μM H_2_DCFDA probe for 30 min at 37 °C in the dark. The cells were then washed, and treated with BO-LLE (1–30 μg/mL), MFs (10 μg/mL) or verbascoside (10 μg/mL) at 37 °C for 10 min. Trolox (100 μM, Tocris) was used as a positive control. Subsequently, UVB at 312 nm, an intensity of 5.45 mW/c^2^, and a dose of 50 mJ/cm^2^ was used to irradiate the cells and incubated further for 30 min at 37 °C in the dark. The fluorescence intensity of intracellular ROS was read using spectrophotometry at 488/530 (exc/em; TECAN infinite M Plex).

### Statistical Analysis

All in vitro experiments were at least conducted three times independently and the expressed values represent the means ± SD. Statistical data analysis was performed using PRISM version 10.2.1 (GraphPad software). Normality was tested using the Shapiro-Wilk test. Statistical significance was determined by two-way Welch ANOVA. Statistical significance was assumed at *p < 0.05, **p < 0.01, ***p < 0.001, ****p < 0.0001.

## Supplementary Material





## Data Availability

The ^1^H NMR data of the microfractions are deposited on Zenodo (DOI: 10.5281/zenodo.13959729).

## Notes

Tables containing ^1^H NMR data of MF21, MF yields, parameter settings for MZmine 3, FBMN, GNPS library search and NAP; additional figures showing MF results in the ROS assay, stacked ^1^H NMR spectra of MF packages, biochemometric molecular network analysis of package III, and TLC overviews of MFs (PDF)
MZmine 3 batch file (XLSX)

## Notes

Dedicated to Dr. Sheo B. Singh, retired from Merck, now with Drew University and Stevens Institute of Technology, for his pioneering work on bioactive natural products.
