# Supplementary material for: Combining the Strengths of MS and NMR in Biochemometrics: A Case Study on Buddleja officinalis
Source: J Nat Prod. 2024 Nov 6;88(5):1099–110. doi: 10.1021/acs.jnatprod.4c00847 (PMC12105003; doi:10.1021/acs.jnatprod.4c00847)
Supplement: Supplementary file 1 [file np4c00847_si_001.pdf]

SUPPORTING INFORMATION

Combining the Strengths of MS and NMR in Biochemometrics: A Case Study on *Buddleja officinalis*

Andreas Wasilewicz,[†,1] *Alexander Areesanan,*[‡,1] *Benjamin Kirchweger,*[†,§] *Sven Nicolay,*[‡] *Eva Waltenberger,*[†] *Mehdi A. Beniddir,*[⊥] *Carsten Gründemann,*[*,‡] *Judith M. Rollinger,*[†] *Ulrike Grienke*[*,†]

[†] Division of Pharmacognosy, Department of Pharmaceutical Sciences, Faculty of Life Sciences, University of Vienna, Josef-Holaubek-Platz 2, 1090 Vienna, Austria

[‡] Translational Complementary Medicine, Department of Pharmaceutical Sciences, University of Basel, Campus Rosental – Mattenstrasse 22, 4058 Basel, Switzerland

[§] Pharmaceutical Biology, Department of Pharmacy, Ludwig-Maximilians-Universität München, Butenandtstraße 5-13, 81377 Munich, Germany

[⊥] Équipe Chimie des Substances Naturelles, BioCIS, CNRS, Université Paris-Saclay, 17 Avenue des Sciences, 91400 Orsay, France

Table of Contents

Figure S1. Stacked ^{1}H NMR spectra (500 MHz, in DMSO-d_6, at 298 K) of packages I (MF19-MF21) and II (MF21-MF25) 3

Table S1. ^{1}H (500 MHz) NMR Spectroscopic Data of Verbascoside and *cis*-Acteoside in DMSO-d_6 (δ in ppm). 4

Figure S2. DPPH assay data of verbascoside, MF21 and MF22 tested at 10 µg/mL. 5

Figure S3. Intracellular ROS measurement of MFs in HCE-T cells. 5

Figure S4. Stacked ^{1}H NMR spectra (500 MHz, in DMSO-d_6, at 298 K) of MF package III (MF28-MF30) referenced to the residual non-deuterated solvent signal (δ_H 2.50 ppm). 6

Figure S5. Molecular network of (A) package II (MF28-MF30) to identify non-cytotoxic compounds in HCE-T cells using the WST-1 assay. 6

Table S2. ^{1}H (500 MHz) and ^{13}C (125 MHz) NMR Spectroscopic Data of Mimengoside A in DMSO-d_6 (δ in ppm). 7

Figure S6. HCE-T cell viability assay of the saponin-enriched fraction. 8

Figure S7. Collective TLCs of MF1-MF34 and BO-LLE. 9

Table S3. Yields of MF1-MF34. 9

Table S4. Methods and Settings used in MZmine 3. 10

Table S5. Parameters used for FBMN, GNPS Library Search and NAP. 11

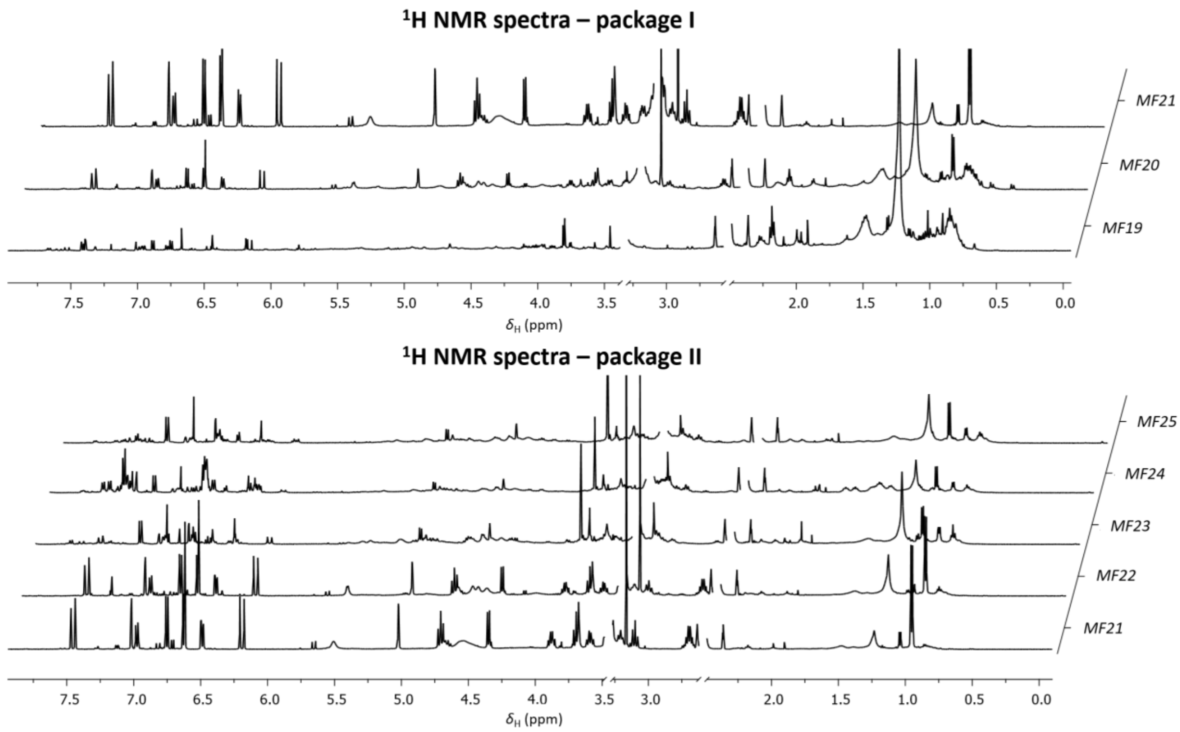

Figure S1. Stacked ^{1}H NMR spectra (500 MHz, in DMSO-d_6, at 298 K) of packages I (MF19-MF21) and II (MF21-MF25) referenced to the residual non-deuterated solvent signal (δ_H 2.50 ppm).

Table S1. ^{1}H (500 MHz) NMR Spectroscopic Data of MF21 identified as Verbascoside and *cis*-Acteoside in DMSO-d_6 (δ in ppm).

position	δ_H, multiplicity (J in Hz)
1	4.34, d (7.86)
2	3.21, t (7.88)
3	3.69, m
4	4.71, t (9.68)
5	3.47, m
6	3.33; 3.37, m
2'	6.62, s
5'	6.49, dd (2.01, 8.07)
6'	6.63, d (5.47)
7'	2.71, m
8'	3.59; 3.87, m
1"	5.02, s
2"	3.67, m
3"	3.34, m
4"	3.10, t (9.40)
5"	3.28, m
6"	0.95, d (6.18)
2"'	7.02, d (2.04)
trans-5"'	6.75, d (8.14)
cis-5"'	6.71, d (8.25)
trans-6"'	6.98, dd (2.03, 8.29)
cis-6"'	7.13, dd (2.14, 8.40)
trans-7"'	7.45, d (15.77)
cis-7"'	6.82, d (13.22)
trans-8"'	6.19, d (15.89)
cis-8"'	5.65, d (12.93)

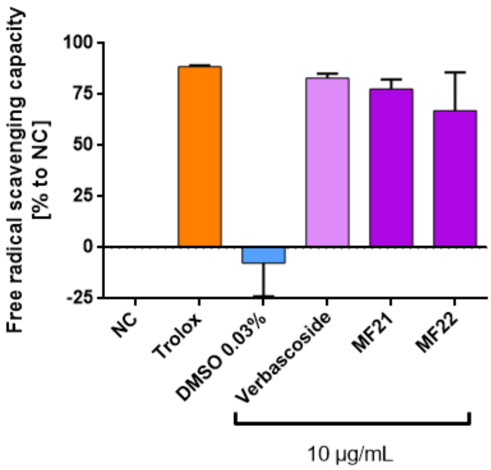

Figure S2. DPPH assay data of verbascoside, MF21 and MF22 tested at 10 µg/mL. The samples were incubated for 30 min with DPPH (100 μM) followed by spectrophotometric measurements to determine their free radical scavenging capacity. Trolox (100 μM) was used as positive control. The data were normalized to the negative control (NC; 0% scavenging capacity) and presented as mean ± standard deviation.

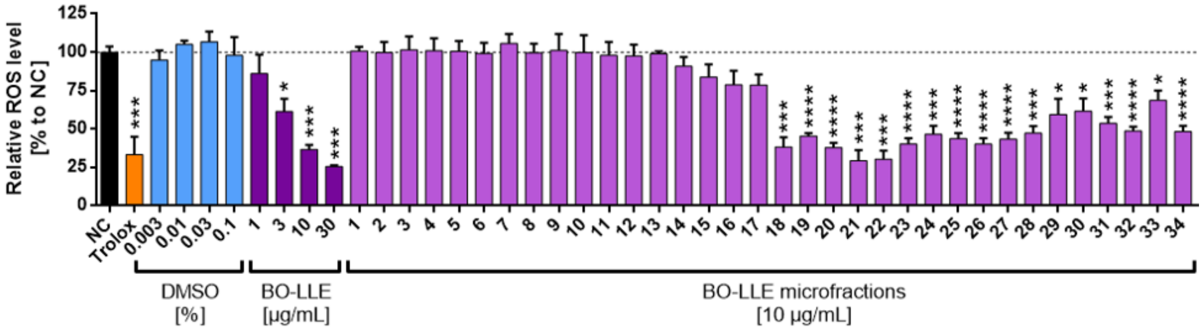

Figure S3. Intracellular ROS measurement of MFs in HCE-T cells. HCE-T were pre-incubated for 30 min with H_2DCFDA (25 μM) prior to the treatment for 10 min followed by UVB exposure. After 30 min, the intracellular ROS fluorescence intensities were measured spectrophotometrically, with Trolox (100 μM) used as a control.

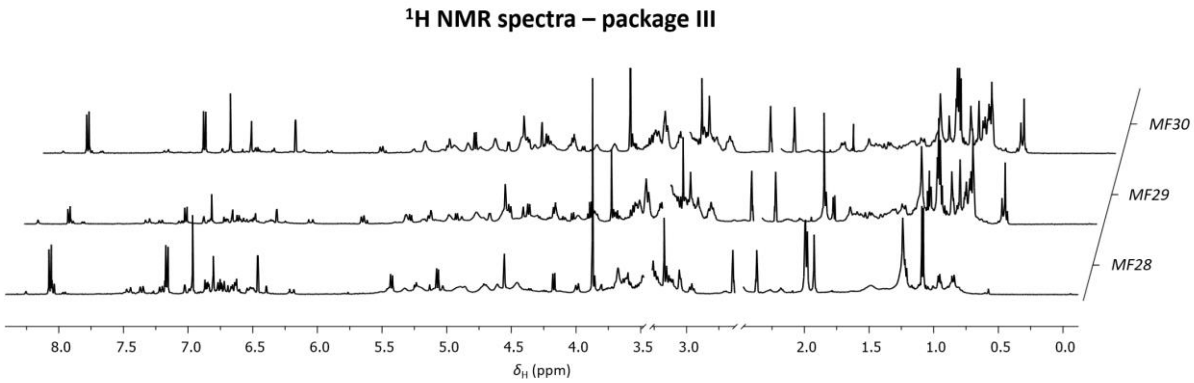

Figure S4. Stacked ^{1}H NMR spectra (500 MHz, in DMSO-d_6, at 298 K) of MF package III (MF28-MF30) referenced to the residual non-deuterated solvent signal (δ_H 2.50 ppm).

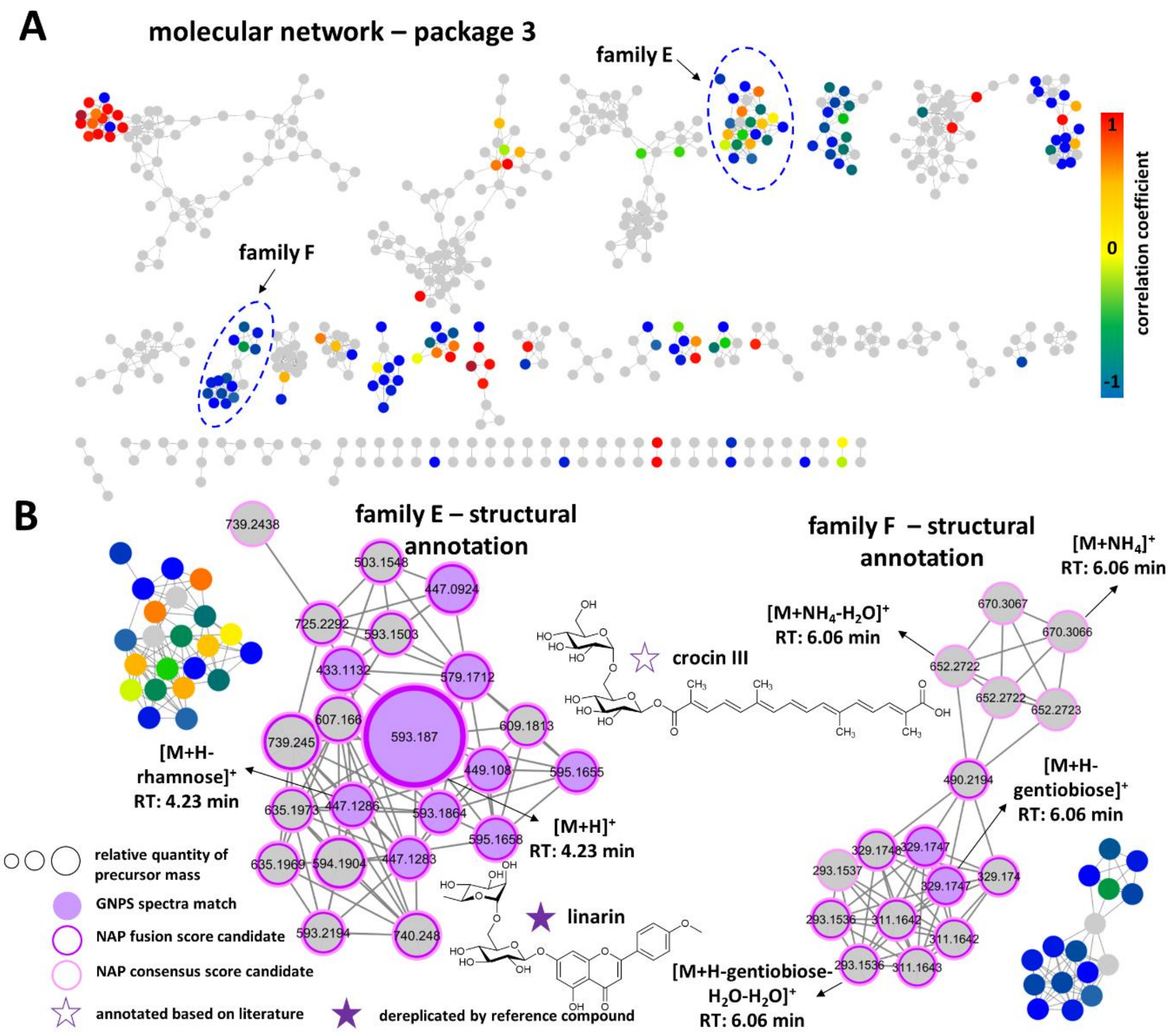

Figure S5. Molecular network of (A) package II (MF28-MF30) to identify non-cytotoxic compounds in HCE-T cells using the WST-1 assay. The color code is based on the correlation coefficient. (B) Structural annotation of negatively correlated nodes, representing linarin in spectral family E and crocin III in spectral family F.

Table S2. ^{1}H (500 MHz) and ^{13}C (125 MHz) NMR Spectroscopic Data of Mimengoside A in DMSO-d_6 (δ in ppm).

position	δ_H, multiplicity (*J* in Hz)	δ_C	δ_C HMBC	δ_H COSY
3	3.46, m	80.4	42.4 (C-4); 11.5 (C-24)	
11	5.27, dd (10.29, 2.47)	131.8		5.79 (H-12)
12	5.79, d (10.54)	131.3	34.9 (C-10); 41.3 (C-8); 83.8 (C-13)	5.27 (H-11)
23	3.04; 3.59, m	62.2		
24	0.59, s	11.6	42.4 (C-4); 45.9 (C5); 62.2 (C-23); 80.4 (C-3)	
25	0.83, s	17.8	overlapped	
26	1.00, s	19.1	41.3 (C-8); 30.6 (C-7); 43.4 (C-14); 52.5 (C-9)	
27	0.89, s	18.8	24.7 (C-16); 41.2 (C-8); 43.4 (C-14)	
28	3.15; 3.58, m	75.9	24.8 (C-22); 41.0 (C-17); 50.6 (C-18); 83.8 (C-13)	3.15 (H-28a); 3.58 (H-28b)
29	0.93, s	34.0	23.2 (C-30); 31.3 (C-21); 34.2 (C-20); 36.3 (C-19)	
30	0.83, s	23.2	overlapped	
Fuc-1	4.31, d (7.85)	102.9	80.4 (C-3)	3.71 (Fuc-H-2)
Fuc-2	3.71, m	74.8	76.1; 82.7 (Glc-C-3); 102.9 (Fuc-C-1)	
Fuc-3	3.59, m	82.7		
Fuc-6	1.10, m	16.5		3.48 (Fuc-H-5)
Glc-1	4.66, m	101.8	74.8 (Fuc-C-2)	
Glc-1'	4.51, m	103.2	82.7 (Fuc-C-3)	
Glc-4'	3.35, m	76.4	100.4 (Rha-C-1)	
Rha-1	4.69, s	100.4	68.5 (Rha-C-5); 70.6 (Rha-C-2); 74.4 (Glc-C-4)	
Rha-6	1.09, m	17.6		3.84 (Rha-H-5)

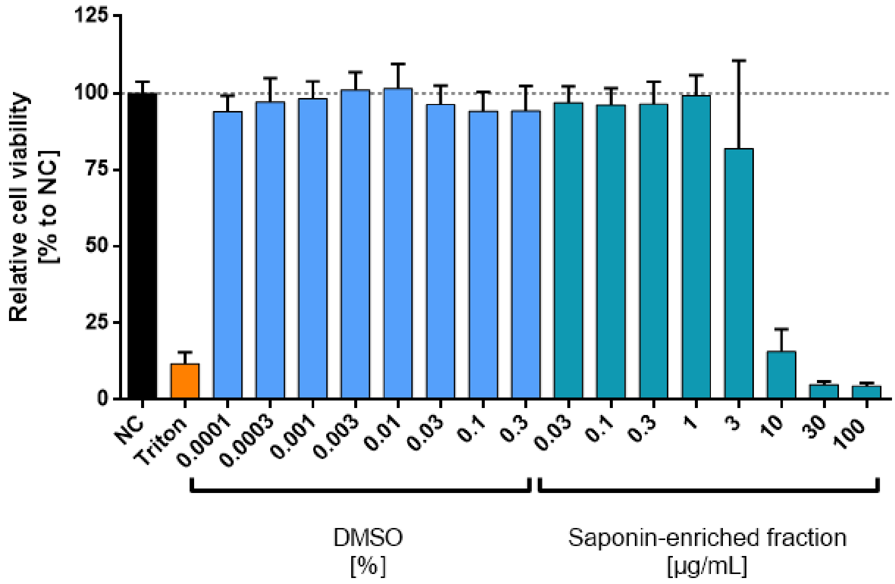

Figure S6. HCE-T cell viability assay of the saponin-enriched fraction. HCE-T cells were treated with the samples for 24 h and the cytotoxicity was determined by colorimetric WST-1 assay. Triton-X 100 (Triton) was used as a control.

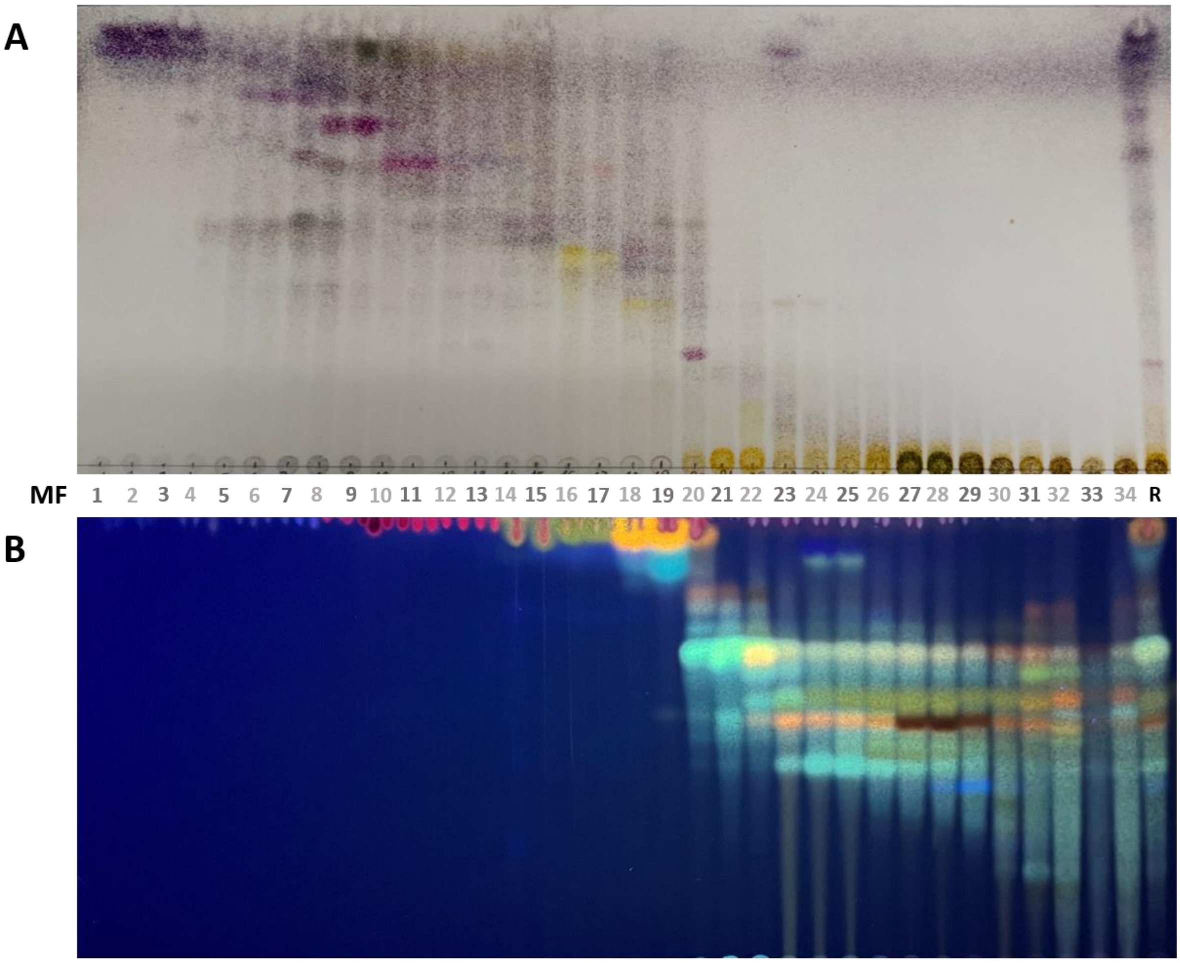

Figure S7. TLC overviews of MF1-MF34 and BO-LLE. (A) MFs analyzed with TLC system 1 (CH_2Cl_2-MeOH-H_2O (9:1:0.1, v/v/v)) at VIS after derivatization with vanillin/sulfuric acid. (B) MFs analyzed with TLC system 2 (EtOAc-H_2O-CH_3COOH-HCOOH (100:26:22:11, v/v/v/v)) at UV_{366} after derivatization with natural product reagent A/PEG400; R = BO-LLE.

Table S3. Yields of MF1-MF34.

MF	Yield [mg]	MF	Yield [mg]	MF	Yield [mg]	MF	Yield [mg]
**1**	296.6	**10**	65.2	**19**	172.5	**28**	29.6
**2**	170.4	**11**	29.3	**20**	410.2	**29**	483.8
**3**	224.7	**12**	144.9	**21**	367.8	**30**	186.6
**4**	87.2	**13**	49.6	**22**	732.3	**31**	225.9
**5**	75.2	**14**	82.1	**23**	376.7	**32**	84.7
**6**	66.1	**15**	86.6	**24**	215.4	**33**	150.3
**7**	195.3	**16**	50.7	**25**	113.2	**34**	94.0
**8**	145.6	**17**	204.5	**26**	114.9		
**9**	43.9	**18**	98.1	**27**	86.0		

Table S4. Methods and Settings used in MZmine 3.

**Mass detection**	
MS1	1.0E+4
MS2	1.0E+1
**ADAP chromatogram builder**	
Minimum consecutive scans	4
Minimum intensity for consecutive scans	5.0E+4
Minimum absolute height	1.0E+5
m/z tolerance	0.002 *m/z* or 10 ppm
**ADAP feature resolver**	
S/N threshold	23
Minimum feature height	5.0E+4
Coefficient/area threshold	10.0
Peak duration range	0.1 – 1.1 min
RT wavelet range	0.0 – 0.08 min
**^{13}C isotope filter**	
m/z tolerance	0.001 *m/z* or 3 ppm
Maximum charge	1
Retention time tolerance	0.003 min
Representative isotope	most intense
**Join aligner**	
m/z tolerance	0.005 *m/z* or 10 ppm
Weight for m/z	3
Retention time tolerance	0.1 min
Weight for RT	1
**Feature list rows filter**	
Minimum aligned features	2
Minimum features in an isotope pattern	2
m/z	100.0000 – 1200.0000
**Peak finder**	
Intensity tolerance	20%
m/z tolerance	0.005 *m/z* or 10 ppm
RT tolerance	0.100 min
Minimum scans	3

Table S5. Parameters used for FBMN, GNPS Library Search and NAP.

**Feature-based molecular networking parameters**	
Quantification table source	MZmine
Parent and fragment ion mass tolerance	0.02 Da
Cosine score	0.6
Network topK	10
Minimum matched fragment ions	4
Maximum connected component size	100
Maximum shift between precursors	500 Da
**GNPS spectra library search**	
Library search minimum matched peaks	6
Score threshold	0.7
Search analogs	enabled
Top results to report per query	3
Maximum analog search mass difference	200 Da
**NAP settings**	
Cosine value to subselect inside a cluster	0.65
N first candidates for consensus score	10
Use fusion result for consensus	enabled
Accuracy for exact mass candidate search	10 ppm
Acquisition mode	positive
Adduct ion type	M+H
Structure database	DNP (internal use with institutional subscription)
Maximum number of candidate structures in the graph	10
Workflow type	MZmine
